# An Ensemble and Multi-View Clustering Method Based on Kolmogorov Complexity

**DOI:** 10.3390/e25020371

**Published:** 2023-02-17

**Authors:** Juan Zamora, Jérémie Sublime

**Affiliations:** 1 Instituto de Estadística, Pontificia Universidad Católica de Valparaíso, Avenida Brasil 2830, Valparaíso 2340025, Chile; 2 ISEP—School of Digital Engineers, 92130 Issy-Les-Moulineaux, France

**Keywords:** clustering, Kolmogorov complexity, multi-view learning, information theory

## Abstract

The ability to build more robust clustering from many clustering models with different solutions is relevant in scenarios with privacy-preserving constraints, where data features have a different nature or where these features are not available in a single computation unit. Additionally, with the booming number of multi-view data, but also of clustering algorithms capable of producing a wide variety of representations for the same objects, merging clustering partitions to achieve a single clustering result has become a complex problem with numerous applications. To tackle this problem, we propose a clustering fusion algorithm that takes existing clustering partitions acquired from multiple vector space models, sources, or views, and merges them into a single partition. Our merging method relies on an information theory model based on Kolmogorov complexity that was originally proposed for unsupervised multi-view learning. Our proposed algorithm features a stable merging process and shows competitive results over several real and artificial datasets in comparison with other state-of-the-art methods that have similar goals.

## 1. Introduction

Multi-source data are a never-ending source of information produced almost in real time by many real-life systems: personal data from social networks, medical data acquired by multiple systems for the same patient, remote sensing images acquired under various modalities, etc. All of these data somehow have to be processed by machine learning algorithms.

However, in the last years, there has emerged a new phenomenon in which machine learning methods themselves have started producing their own multiple representations of the same data mainly due to the explosion in the number of algorithms and, in particular, deep learning algorithms that extract features from data. For instance, in the field of natural language processing, text and speech data can be analyzed and clustered from widely different representations and features, and there is, therefore, a need to reconcile and somehow merge these results [1,2]. The same problem exists in many other domains, such as image processing, where different architectures of convolutional neural networks may extract different features and representations. However, this is particularly problematic in the context of unsupervised learning when there is no supervision to decide which representations are the best, and when the only solution is often to produce clustering based on the various possible representations, and thus to merge them all the while solving conflicts. Furthermore, this unsupervised process also has to detect and discard low-quality and noisy representations.

Whether the multiple representations are native to the data or produced artificially by machine learning algorithms, the unsupervised exploration of multi-view data can be regrouped under the terms of multi-view clustering [3] when dealing with multiple representations of the same objects or cluster ensembles [4] when dealing with several partitions of the same objects by multiples algorithms. In this work, we deal with a multi-view application where the data have multiples representations, but we use methods based on the fusion of partitions that are very similar to ensemble learning problems. To tackle such a multi-view clustering problem, two types of approaches exist: The first one consists in attempting a global clustering of the multi-view system using an algorithm that has access to all the views. The second one consists in running algorithms locally in each view and then finding a solution to merge the partitions into a global result.

In this paper, we consider the second approach, which allows for the selection of local algorithms better adapted to each view-specific data representation, and we propose a novel merging method that aims at the fusion of various clustering partitions. Our method uses information theory and the principle of minimum description length [5,6] to detect points of agreements as well as conflicts between the local partitions, and features an original method to reduce these conflicts as the partitions are merged. We call our method KMC for “Kolmogorov-based multi-view clustering”.

This idea was successfully used in earlier work about multi-view clustering without merging partitions [7] and for text corpus analysis [8]. This work brings the following novel aspects and contributions:

1. Our main scientific contribution is the proposal of a new heuristic method relying on Kolmogorov complexity to merge partition in an unsupervised ensemble learning context applied to multiview clustering. Compared with earlier methods, we remove the reliance on an arbitrary pivot to choose the merging order. Instead, we reinforce the use of Kolmogorov complexity to make the choice of the merging order, thus rendering our algorithm deterministic, while earlier versions and methods were not. Our method also explores more of the solution space, thus leading to better results.
2. We propose a large comparison of unsupervised ensemble learning methods—including four methods from the state of the art—in a context which is not restricted to text corpus analysis, both in terms of state-of-the-art methods but also datasets.
3. While not a scientific or technical contribution (because our method relies on known principles), our algorithm brings some novelty in the field of unsupervised ensemble learning, where no other method relies on the same principle. We believe that such diversity is useful to the field of clustering, where a wider choice of methods is a good thing because of the unsupervised context.

Finally, while it is not a technical or scientific contribution, we analyze the effects of various levels of noise in different number of views, and the effect of changing the number of clusters. We assess how these parameters affect the performance of our proposed method in terms of result quality. These results, while linked to our proposed methods, may shed some light on the behavior of other methods in the same context.

This paper is organized as follows: In Section 2, we present some of the main methods and approaches both for multi-view clustering and partition fusion methods based on various principles. Section 3 introduces our proposed algorithms. Section 4 features our experimental results and some comparisons with other methods. Finally, in Section 5, we give some conclusions as well as some insights as to what future improvements and works could be performed based on our proposal.

## 2. State of the Art

The problem of multi-view clustering is relatively common in unsupervised learning and has been tackled from different angles depending on the intended application. The most common method is to use a global function over all views and to merge all partitions. Several such methods will be presented in this state of the art, where we will also discuss their pros and cons.

Let us begin by presenting the different terminologies used for multi-view approaches and what they entail [9]:

- Multi-view clustering [2,3,10,11,12,13,14,15,16,17,18,19,20,21] is concerned with any kind of clustering, where the data are split into different views. It does not matter whether the views are physically stored in different places, and if the views are real or artificially created. In multi-view clustering, the goal can either be to build a consensus from all the views, or to produce clustering results specific to each view.
- Distributed data clustering [22] is a sub-case of multi-view clustering that deals with any clustering scenario where the data are physically stored in different sites. In many cases, clustering algorithms used for this kind of task will have to be distributed across the different sites.
- Collaborative clustering [23,24,25,26,27] is a framework in which clustering algorithms work together and exchange information with the goal of mutual improvement. In its horizontal form, it involves clustering algorithms working on different representations of the same data, and it is a sub-case of multi-view clustering with the particularity of never seeking a consensus solution but rather aiming for an improvement in all views. In its vertical form, it involves clustering algorithms working on different data samples with similar distributions and underlying structures. In both forms, these algorithms follow a two-step process: (1) A first clustering is built by local algorithms. (2) These local results are then improved through collaboration. A better name for collaborative clustering could be model collaboration, as one requirement for a framework to qualify as collaborative is that the collaboration process must involve effects at the level of the local models.
- Unsupervised ensemble learning, or cluster ensembles [28,29,30,31,32,33,34,35,36] is the unsupervised equivalent of ensemble methods from supervised learning [37]: It is concerned with either the selection of clustering methods, or the fusion of clustering results from a large pool, with the goal of achieving a single best-quality result. partitions. This pool of multiple algorithms or results may come from a multi-view clustering context [38], or may just be the unsupervised equivalent of boosting methods, where one would attempt to combine the results of several algorithms applied to the same data. Unlike collaborative and multi-view clustering, ensemble clustering does not access the original features, but only the crisp partitions.

In this paper, collaborative clustering and distributed clustering are not considered. We focus solely on the problem of multi-view and ensemble clustering: we merge clustering partitions no matter their origin and without accessing the original features. Our problem is therefore similar to the one introduced by Strehl and Ghosh in their paper [28], where they present the problem of combining multiple partitions of a set of objects without accessing the original features.

We will now review some of the works that are the most closely related to our proposed method. A more extensive survey of cluster ensemble methods can be found in [36].

In [2], the authors propose a multi-view clustering method applied to text clustering when texts are available under multiple representations. Their method is very similar to [19] in the way that they attempt at merging the different partitions: First, similarity matrices are computed in three different ways, namely, two based on partition memberships and another one based on feature similarity. Then, a combined similarity matrix is obtained from those three previous ones, and a standard clustering technique is applied to produce the consensus partition.

In [39], the authors address the problem of large-scale multi-view spectral clustering. They do so using local manifolds fusion to integrate heterogeneous features based on approximations of the similarity graphs.

In [40], a similar method is proposed for partition fusion in a multi-view clustering context. It also relies on a graph-based approach, with the addition of a weight system to account for the clustering capacity differences of the views.

In [41], the authors address partial multi-view clustering, a specific case of multi-view clustering where not all data are in all views. They uses latent representations and seek the closest available data when one is missing in a view.

In [21], the authors address the issue of feature selection in multi-view clustering. They propose a global objective function (quite similar to the ones found in collaborative clustering) in which each feature of each view is automatically weighted to ensure smooth convergence. In the original paper, the authors adapted this method for multi-view K-Means.

In [42], the authors propose a graph based multi-view clustering method which merges the data graphs of all views. It weights the views and detects the number of clusters in an automated manner.

In [43], the authors tackled the problem of multi-view clustering under the assumption that each view or each partition can be seen as a perturbation of the consensus clustering, and that it is possible to weight them so that the partitions closer to the consensus are more important. They do so by using subspace clustering and graph learning in each view.

Another consensus generation strategy is proposed in [44], where a co-association matrix is built from the ensemble partitions and then it is improved by removing low coefficients called negative evidences. This removal procedure is performed in conjunction with a N-Cut clustering in multiple rounds, and finally the best partition is reported.

Based on an initial cluster similarity graph, ref. [45] proposed an enhanced co-association matrix that allows to simultaneously capture the object-wise co-occurrence relationships as well as the multi-scale cluster-wise relationship in ensembles. Finally, two consensus criteria are proposed, namely hierarchical and meta-cluster-based functions. Ref. [46] proposed a randomized subspace generation mechanism to build multiple-base clusterings. From these partial solutions, an entropy weighted combination strategy is applied in order to obtain an enriched co-association matrix that serves as a summary of the ensemble. Finally, they employed three independent consensus solutions over the co-association matrix, namely a hierarchical clustering, a bipartite graph clustering and a spectral clustering.

Finally, we can mention the work of Yeh and Yang [47], which is very relevant to understanding the difficulty of properly evaluating ensemble clustering methods, and where the authors propose a fuzzy generalized version of the Rand Index for ensemble clustering.

As one can see, all these recent algorithms for partitions fusion are actually built so that they are not so much a merging method of existing partitions, but rather global clustering frameworks that seek and merge partitions in all views at the same time. While the end goal is the same as our proposed method—finding a consensus clustering partition—our method is different in the sense that it starts from existing partitions and has no access to the original data features. As depicted in the experimental section, this key difference can make our method difficult to compare in a fair manner with the above described works from the state of the art.

One of the strong points of our method is that it is ensemble clustering, in the sense that it combines pre-existing clustering partitions, but it is also multi-view clustering since these partitions come from different sets of features or multiple views. As a consequence, the flexibility on the algorithms we can use over local data views is a distinctive characteristic regarding classical multi-view clustering, where the same clustering method does it all from the local views. However, it can be costly in terms of performance, especially if the local algorithms are not state of the art.

## 3. The Proposed Method

### 3.1. Problem Definition and Notations

Let us consider a data space X, which can be decomposed into *M* views so that $X=X^{1}\times \ldots \times X^{M}$, where the *M* spaces $X^{i}$, that may or may not overlap depending on the application. The spaces $X^{i}$ will therefore be the spaces associated to the views. The interdependence between the views is not solely contained in the definition of the different views $X^{j}$, but also in the probability distribution *P* over the whole space X.

Let $X=\{x_{1},x_{2}\ldots ,x_{N}\}$, X∈X be a set of *N* objects split into the *M* views. We note the local views of these data $X^{1}$ to $X^{M}$ ($\forall i\;X^{i}\in X^{i}$). As such, any view $X^{i}$—the realization of the dataset over $X^{i}$—is a matrix containing *N* lines and the columns (attributes) attached with view $X^{i}$. From there, $x_{n}^{i}$ will denote the *n*-th line of view *i*, and is a vector.

Let $\Pi =\{\Pi^{1},\Pi^{2},\ldots ,\Pi^{M}\}$ be a set of crisp partitions of objects in *X* computed over the *M* views. Each local partition $\Pi^{i}$ is a vector of size *N* which to any data $x_{n}^{i}\in X^{i}$ associates a hard cluster $K_{a}^{i}$, $a\in [1\ldots K^{i}]$. Since different views can have different numbers of clusters, we note $K^{i}$ (without lower index), the number of cluster in any view *i*. For simplification purposes, $K_{K^{i}}^{i}$, the last cluster of any view *i*, will simply be noted $K^{i}$. From there, the association function $L^{i}\left(x\right)$ is defined as follows:

(1) $$L^{i}:X^{i}\to \left[1\ldots K^{i}\right]$$

In other words, the function $L^{i}$ maps any element of view *i* to a cluster of the same view. It is the result of a clustering method applied to view *i*. From there, we have that each local partition $\Pi^{i}$ can be written as follows: $\Pi^{i}=\left\{L^{i}\left(x_{1}\right),L^{i}\left(x_{2}\right),\ldots ,L^{i}\left(x_{N}\right)\right\}$. Please note that we write $L^{i}\left(x_{n}\right)$ to simplify the notations, as the view is implied in the mapping function index, but the proper notation would be $L^{i}\left(x_{n}^{i}\right)$.

Like many works in multi-view clustering, in order to measure the overlap between clusters in different partitions, we use a confusion matrix [48]. For two views *i* and *j*, this matrix which we note $\Omega^{ij}$ is of size $K^{i}\times K^{j}$ and defined as follows:

(2) $$\Omega^{ij}=\left(\begin{array}{ccc} \omega_{1,1}^{ij} & \cdots & \omega_{1,K^{j}}^{ij}\\ \vdots & \ddots & \vdots \\ \omega_{K^{i},1}^{ij} & \cdots & \omega_{K^{i},K^{j}}^{ij} \end{array}\right)\;\mathrm{where}\;\omega_{a,b}^{ij}=\frac{|K_{a}^{i}\cap K_{b}^{j}|}{|K_{a}^{i}|}$$

In other words, each $\omega_{a,b}^{ij}$ measures the percentage of elements that belong to cluster $K_{a}^{i}$ in view *i* that belong to cluster $K_{b}^{j}$ in view *j*. Please remember that $\Omega^{ij}$ maps from view *i* to view *j* and that this mapping may be different from the mapping acquired from $\Omega^{ji}$, especially if the two views have different numbers of clusters: there is no symmetry hypothesis here.

From there, for each cluster in each view, it is possible to find the maximum agreement cluster in any other view simply by searching the maximum value in each of the lines of the corresponding matrix $\Omega^{ij}$. Let us note $\Phi^{j}\left(K_{a}^{i}\right)$, the maximum agreement cluster in partition $\Pi^{j}$ for cluster $K_{a}^{i}$ of partition $\Pi^{i}$:

(3) $$\Phi^{j}\left(K_{a}^{i}\right)=\underset{b\in [1..K^{j}]}{\mathrm{argmax}}\omega_{a,b}^{ij}$$

Lastly,

(4) $$K_{\cdot }^{i}\left(x\right)=\left\{x^{\prime }\in X|L^{i}\left(x^{\prime }\right)=L^{i}\left(x\right)\right\}$$

#### Table with All Notations

Table 1 below contains all notations that will be used in the algorithm presented in the next sections. Some of these notations have already been presented with details and equations; others will be detailed more as the different concepts and algorithms are presented.

### 3.2. Merging Partitions Using Kolmogorov Complexity

In the work of [5,6], the notion of minimum description length (MDL) is introduced, with the *description length* being the minimal number of bits needed by a Turing machine to describe an object. This measure of the minimal number of bits is also known under the name Kolmogorov complexity.

If M is a fixed Turing machine, the complexity of an object **x** given another object **y** using the machine M is defined as $K_{M}\left(x|y\right)=\min_{p\in P_{M}}\left\{l(p):p(y)=x\right\}$, where $P_{M}$ is the set of programs on M, p(y) designates the output of program *p* with argument *y* and *l* measures the length (in bits) of a program. When the argument y is empty, we use the notation $K_{M}\left(x\right)$ and call this quantity the complexity of x. The main problem with this definition is that the complexity depends on a fixed Turing machine M. Furthermore, the universal complexity is not computable since it is defined as a minimum over all programs of all machines.

In relation to this work, in [7], the authors solved the aforementioned problem by using a fixed Turing machine before applying this notion of Kolmogorov complexity to collaborative clustering, which is a specific case of multi-view clustering, where several clustering algorithms work together in a multi-view context but aim at improving each other’s partitions rather than merging them [23]. While collaborative clustering does not aim at a consensus, this application is still very close to what we try to achieve in this paper, where we try to merge partitions of the same objects under multiple representations. For these reasons, we decided to use the same tool.

In the rest of this paper, just as the authors did in [7], we will consider that the Turing machine M is fixed, and to make the equations easier, we will denote by K(x) the complexity of x on the chosen machine. Then, we adapt the equations used in their original paper to our multi-view context for text mining and we use Kolmogorov complexity as a tool to compute the complexity of one partition given another partition. The algorithm to do so and how we use it is described in the next section.

### 3.3. The KMC Algorithm

**Figure 1:**
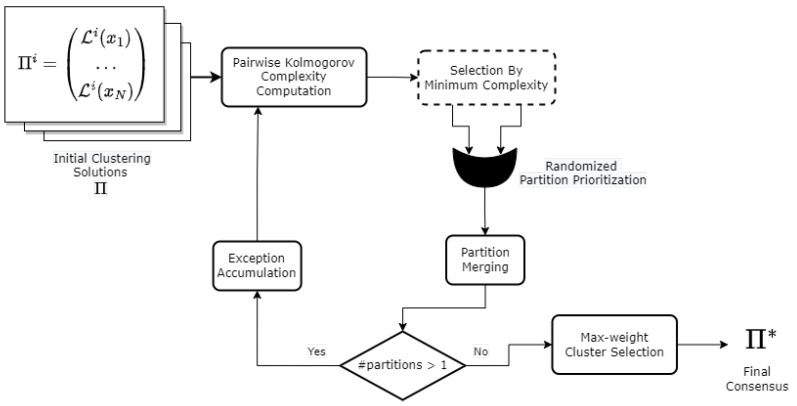
Overall scheme of the KMC method to produce the consensus partition $\Pi^{*}$.

Our goal is to combine several partitions in order to build a final consensus. To this end, we perform successive pairwise fusion procedures between partitions following a bottom-up strategy until we reach a single soft partition. Subsequently, the consensus is generated by picking the cluster with the maximum weight for each data point. Figure 1 depicts an overall scheme of the proposed method, and Algorithms 1 and 2 show a detailed description for the two procedures that make up the proposal.

Let us consider that for a set Π of initial partitions, there are $O(|\Pi {|}^{2})$ candidate pairs to merge. In order to overcome the combinatory explosion, we will use a greedy criteria to pick a pair of partitions: the Kolmogorov complexity of partition $\Pi^{i}$ knowing partition $\Pi^{j}$ is computed by following the procedure described in [7] as shown in Equation (5) below:

(5) $$K\left(\Pi^{i}|\Pi^{j}\right)=K^{j}\times \left(\log K^{i}+\log K^{j}\right)+\left|\epsilon_{i,j}\right|\times \left(\log n+\log K^{i}\right)$$

In Equation (5), $K^{i}$ still denotes the number of clusters in a given partition *i* as defined earlier, and $|\epsilon_{i,j}|$ is the number of errors in the mapping from partition *i* to partition *j*. These errors correspond to data that do not adhere to the maximum agreement partition mapping $\Phi^{\left(j\right)}$, increasing the overall complexity and are also likely to cause issues when merging the partitions:

(6) $$\epsilon_{i,j}=\left\{x\in X|\forall p\in \left[1..K^{i}\right],L^{i}\left(x\right)=K_{p}^{i},\;L^{i}\left(x\right)\neq \Phi^{j}\left(K_{p}^{i}\right)\right\}$$

**Figure 2:**
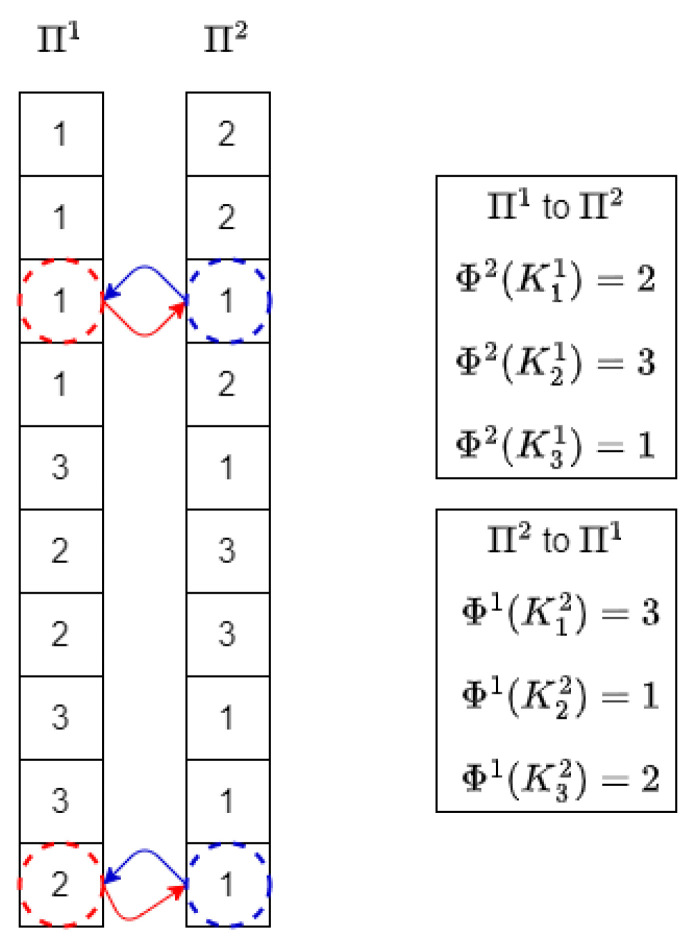
Finding the mapping errors based on the majority rules for 2 partitions with 10 objects and 3 clusters per partition. Enclosed in a dashed red line are the objects identified as mapping errors for the first partition, and in a dashed blue line those identified for the second partition.

Please note that these errors as defined in Equation (6) can be computed simply by browsing through the partitions and based on the majority rules, as shown in Figure 2.

#### 3.3.1. Overall View of the Main Procedure

Algorithm 1 unfolds as follows for each round: First the pair of partitions with the least complexity based on Equation (5) is selected as described in Line 5. The pair of partitions with the least complexity value is selected as described in Line 5 of Algorithm 1. In Lines 9 and 11, the merge procedure is called. It is worth mentioning that since the commutative property does not hold for this operation, in the original version of the algorithm, a randomized criterion inspired by the farthest-first traversal approach presented in [49] was employed in Line 8 to pick the first argument taken by this call. In the current version, for each input partition, the Kolmogorov complexity is computed against several random partitions, and the one with the highest average complexity among the two input partitions is chosen as the first argument for this function. The rationale behind this decision is that a more discordant input partition with respect to several random configurations has more information contained than another one more agreeable. After all partitions have been merged into a single one at Line 15, the last set of exceptions is processed and used in order to compute the final label for each object (see Lines 17–19).
**Algorithm 1:** Main procedure for building the consensus partition.
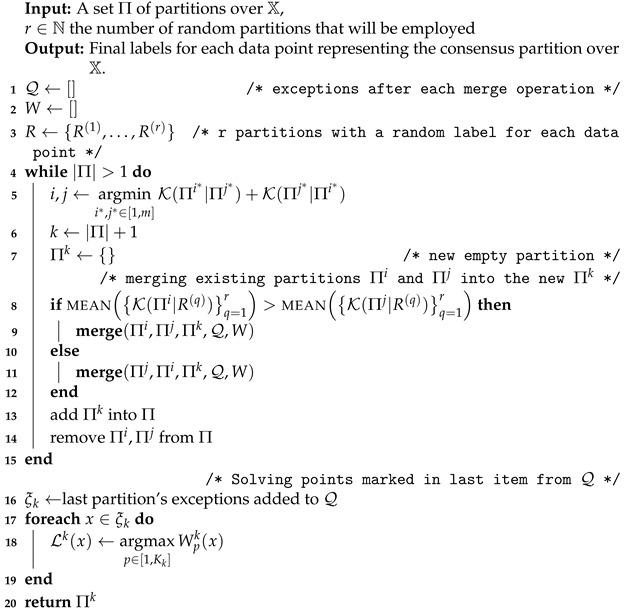


#### 3.3.2. Merging Two Views/Partitions

Regarding the merge operation between two partitions described in Algorithm 2, when it is performed between two partitions $\Pi^{i}$ and $\Pi^{j}$, each cluster in $\Pi^{i}$ is combined with its maximum agreement cluster in $\Pi^{j}$ (computed as shown in Figure 2). Let $\Pi^{k}$ denote the new partition produced from the merging of these two clusters. First, for all clusters in $\Pi^{i}$, the majority clusters in $\Pi^{j}$ are listed for a posterior fusion. Additionally, clusters in $\Pi^{j}$ are also added to the lists of their majority clusters in $\Pi^{i}$. After that, in Lines 4–6, all the objects with total agreement between each pair of merged clusters are put together in the same cluster of the new partition $\Pi^{k}$.

Since these successive partition fusions are performed by following the maximum agreement criteria between clusters as stated in Equation (3), it is likely that some data points—identified as mapping errors in their original partitions as per Equation (6)—will not fit to this rule and hence be marked as exceptions that shall be dealt with at the final stage and put in a specific subset during the execution of the subsequent merge operations. As defined by Equation (6) the exception set $\xi_{k}\left(=\epsilon_{i,j}\right)$ for the newly created partition $\Pi^{k}$ is made up by objects whose cluster in the first former partition does not match the majority rule cluster in the second former partition. Algorithm 2 addresses this task in Lines 8–11. Finally, we store a history of all previous exceptions in list Q.
**Algorithm 2: Merge** procedure that fuses two partitions into a new one identifying also problematic points as exceptions.
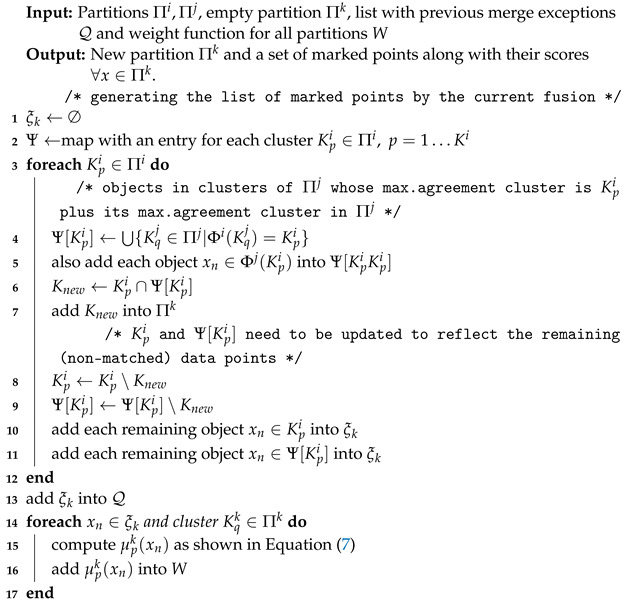


#### 3.3.3. Handling Mapping Errors through the Merge Process

The objects marked as exceptions have no crisp cluster membership within the new partition. Therefore, these mapping errors generated after the fusion of $\Pi^{i}$ with $\Pi^{j}$ will have an uncertain cluster membership in the newly generated $\Pi^{k}$. These memberships will be settled/decided at the final stage of the method when the last partition is produced. As a means to counteract this ambiguity, we measure the support of a data object *x* in each cluster *p* of $\Pi^{k}$ by a function of the similarity/closeness between this cluster and the clusters $L^{i}\left(x\right)$ and $L^{j}\left(x\right)$ within which this data object was grouped in the former partitions $\Pi^{i}$ and $\Pi^{j}$ respectively. For each object previously contained either in $\xi_{i}$ or $\xi_{j}$, its support is a function of the similarity/closeness between cluster $p\in [1\ldots K^{k}]$ and its maximum agreement cluster in $\Pi^{i}$ or $\Pi^{j}$ correspondingly.

Formally, for any merge operation and without loss of generality, let $\Pi^{i}$ and $\Pi^{j}$ be the two partitions/views whose fusion produces $\Pi^{k}$, and let J(U,V) be the classical Jaccard similarity between two sets *U* and *V*. For every data point $x\in \xi_{k}$, its membership in a cluster $K_{p}^{k}$ ($p\in [1\ldots K^{k}]$) is denoted by $\mu_{p}^{k}\left(x\right)$ and defined as follows:

(7) $$\mu_{p}^{k}\left(x\right)=\left\{\begin{array}{cc} \frac{1}{2}\left[J\left(K_{p}^{k},L^{i}\left(x\right)\right)+J\left(K_{p}^{k},L^{j}\left(x\right)\right)\right] & x\notin \xi_{i}\;\mathrm{and}\;x\notin \xi_{j}\\ \frac{1}{2}\left[\mu_{\Phi^{i}\left(K_{p}^{k}\right)}^{i}\left(x\right)\cdot J\left(K_{p}^{k},\Phi^{i}\left(K_{p}^{k}\right)\right)+J\left(K_{p}^{k},L^{j}\left(x\right)\right)\right] & x\in \xi_{i}\;\mathrm{and}\;x\notin \xi_{j}\\ \frac{1}{2}\left[J\left(K_{p}^{k},L^{i}\left(x\right)\right)+\mu_{\Phi^{j}\left(K_{p}^{k}\right)}^{j}\left(x\right)\cdot J\left(K_{p}^{k},\Phi^{j}\left(K_{p}^{k}\right)\right)\right] & x\notin \xi_{i}\;\mathrm{and}\;x\in \xi_{j}\\ \frac{1}{2}\left[\mu_{\Phi^{i}\left(K_{p}^{k}\right)}^{i}\left(x\right)\cdot J\left(K_{p}^{k},\Phi^{i}\left(K_{p}^{k}\right)\right)+\mu_{\Phi^{j}\left(K_{p}^{k}\right)}^{j}\left(x\right)\cdot J\left(K_{p}^{k},\Phi^{j}\left(K_{p}^{k}\right)\right)\right] & x\in \xi_{i}\;\mathrm{and}\;x\in \xi_{j} \end{array}\right.$$

Equation (7) states the four scenarios that may be found when computing the membership weights for the mapping errors:

- First, a data object could be identified as an exception to the majority rule of the current merge operation as formalized in the first case of Equation (7).
- The next two cases show the scenarios in which the data object could come from errors generated in prior merging stages in either of the two former partitions, but not in both.
- The final case defined in Equation (7) is distinguished from previous definitions by denoting the scenario where the data object has been dragged from mapping errors in both input partitions.

The rationale behind Equation (7) is twofold: First, the higher the agreement between a cluster in the new partition and the object cluster in the source partition, the higher the weight value. Second, when no cluster information is available for the object in the source partition, the maximum agreement information is employed, and its value is weighted by the cluster support in the source partitions $\Pi^{i}$ and $\Pi^{j}$. Finally, the overall support for object *x* in cluster *p* is expressed by the mean value between the supports of both input partitions.

As a final remark about the operation of the proposed method, it is important to indicate that once a point is marked as an exception, it remains so through all the subsequent fusions and also that all the exceptions are solved only at the end of the complete merging process. After all the views are subsequently fused into a single partition, every data point has a score greater or equal than zero for each cluster. This carry-over strategy of weighted membership coefficients enables the resolution of the cluster assignment problem for the discordant data objects at the final stage of Algorithm 1 in Line 17. At this point, it is possible to obtain a consensus by picking the cluster with the maximum weight for each data point. The bottom right part of Figure 1 sketches this part of the process until the final consensus is obtained.

### 3.4. Computational Complexity: Discussion

The overall complexity and computation time of an ensemble clustering method is often difficult to assess, as it depends on both the complexity of the clustering methods used in the different views to create the original partitions (which may be paralleled or not), and also on the ensemble process to merge the partitions.

In the case of our KMC algorithm, given *M* views with *N* elements and a maximum of *K* clusters per view, the cost to map all clusters and errors between views is in $O(M^{2}KN)$. We can have a maximum of log(M) merges, which gives us a total complexity in $O(M^{2}\log\left(M\right)KN)$ for the ensemble learning part. In most cases, *K* should be negligible compared to *N*. Which quantity might be the most important between $M^{2}\log\left(M\right)$ and *N* can be up for discussion depending on the number of views *M*. Still, we believe that in most cases, the number of lines *N* is the dominant quantity. Furthermore, if the clustering methods used to generate the original partitions have a complexity beyond linear—in $O(N^{2})$ or $O(N^{2}\log N)$ for instance, as can be possible with several clustering methods other than K-means or a Gaussian mixture model—then the complexity of our multi-view ensemble learning method KMC would be negligible anyways compared to that of the original algorithms used to create the initial partitions.

## 4. Experimental Analysis

Throughout this and the following sections, the name of the proposed method is KMC.

### 4.1. Clustering Measures

To assess the quality of the clustering consensus, we employ the following external measures: entropy, purity and normalized mutual information [28]. Given a method M, a partition $\Pi^{M}$ built after its execution and the gold standard partition $\Pi^{T}$:

Following [50], entropy measures the amount of class confusion within a cluster. The lower its value, the better the clustering solution. Thus, it is defined as

(8) $$\mathrm{Entropy}\left(\Pi^{M},\Pi^{T}\right)=\sum_{p=1}^{K^{M}}-\frac{|K_{p}^{M}|}{N\log K^{T}}\left(\sum_{q=1}^{K^{T}}\frac{|K_{p}^{M}\cap K_{q}^{T}|}{|K_{p}^{M}|}\log\left(\frac{|K_{p}^{M}\cap K_{q}^{T}|}{|K_{p}^{M}|}\right)\right)$$

The purity of the partition proposed by the method M is defined as the number of correctly assigned objects, where the majority class is set as the label for each cluster. This is as follows:

(9) $$\mathrm{Purity}\left(\Pi^{M},\Pi^{T}\right)=\frac{1}{N}\sum_{p=1}^{K^{M}}\max_{j}\left|K_{p}^{M}\cap K_{j}^{T}\right|$$

Normalized mutual information measures the level of agreement between a partition produced by a method and a ground truth partition also correcting the bias induced by the non-normalized version of this measure when the number of clusters increases. It is defined as follows:

(10) $$\mathrm{NMI}\left(\Pi^{M},\Pi^{T}\right)=\frac{\sum_{p=1}^{K^{M}}\sum_{q=1}^{K^{T}}\frac{|K_{p}^{M}\cap K_{q}^{T}|}{N}\log\frac{N|K_{p}^{M}\cap K_{q}^{T}|}{|K_{p}^{M}\left|\right|K_{q}^{T}|}}{0.5\left(-\sum_{p=1}^{K^{M}}\left(\frac{|K_{p}^{M}|}{N}\right)\log\left(\frac{|K_{p}^{M}|}{N}\right)-\sum_{q=1}^{k^{T}}\left(\frac{|K_{q}^{T}|}{N}\right)\log\left(\frac{|K_{q}^{T}|}{N}\right)\right)}$$

### 4.2. Analysis on Real Data and Comparison against Other Ensemble Methods

In order to further validate the performance of our proposed method, we assessed its results against three state-of-the-art methods over the three above-mentioned clustering measures. Additionally, we report the results on nine publicly available datasets generated from 3Sources (Available from http://mlg.ucd.ie/datasets/3sources.html, accessed on October 2022), BBC (3), BBCSports (3) (both available from http://mlg.ucd.ie/datasets/segment.html, accessed on October 2022), Handwritten digits (Available from http://archive.ics.uci.edu/ml/datasets/Multiple+Features, accessed on October 2022) and Caltech (Available from https://github.com/yeqinglee/mvdata, accessed on October 2022). In order to set a fair evaluation environment, for all datasets, a single set of base partitions was generated for each data view by using the Cluto toolkit (Code available at http://glaros.dtc.umn.edu/gkhome/cluto/cluto/overview, accessed on August 2022).

The **3Sources** dataset was collected from three well-known online news sources: BBC, Reuters, and The Guardian. A total of 169 articles were manually annotated with one or more of the six topical labels: business, entertainment, health, politics, sport, and technology.

The **BBC** and **BBCSports** data were collected from BBC-news, and originally **BBC** contained 2225 documents annotated into 5 topics, while **BBC-Sports** comprised 737 documents also with 5 annotated labels. Following [13], from each corpus 2–4 synthetic views were constructed by segmenting the documents according to their paragraphs. Therefore, for each one, 3 multi-view datasets are used, namely BBC-seg2, BBC-seg3 and BBC-seg4 with 2, 3 and 4 views, respectively. The same idea applies for BBCSports-seg2, BBCSports-seg3 and BBCSports-seg4.

The **Handwritten** digits data contain 2000 instances for ten digit classes (0–9), and the views are built from six subsets of features: 76 Fourier coefficients of the character shapes, 216 profile correlations, 64 Karhunen–Loeve coefficients, 240 pixel averages, 47 Zernike moments and 6 morphological features.

**Caltech** is a dataset consisting of 2386 images grouped in 20 categories. We follow [39] and use six groups of handcrafted features as views, namely Gabor features, wavelet moments, CENTRIST features, HOG features, GIST features and LBP features.

For each dataset, only the documents with labels in all views are used. The details for each collection are presented in Table 2.

#### 4.2.1. Baseline Methods

We compare the performance results of our KMC algorithm against eight baseline methods: three approaches originally proposed by Strehl and Ghosh [28], namely cluster-based similarity partitioning (CSPA), hyper graph partitioning (HGPA) and meta clustering (MCLA) (Matlab code for these three methods available at http://strehl.com/soft.html, accessed on July 2022). ECPCS (ensemble clustering via fast propagation of cluster-wise similarities) with its two variants from [46] and MDEC (multidiversified ensemble clustering) with its three variants from [45]. For all these methods, their parameters were set as suggested in their corresponding papers.

#### 4.2.2. Operational Details of the Compared Methods

In order to run Algorithm 1, the number of random partitions was set to 80. Additionally, KMC was executed 10 times over each dataset, and the average clustering quality of the final consensus was reported. Considering that we used external performance criteria and that the methods under evaluation need the number of final clusters as an input parameter, the number of clusters was always set to the number of ground truth classes.

#### 4.2.3. Discussion of the Experimental Results

Table 3 shows the results obtained by each method over each dataset. The best result for each measure on each dataset is highlighted in bold font, and the second best is underlined.

Overall, the proposed method attains competitive results over several datasets of varying sizes: In seven out of nine datasets, the consensuses built by our KMC algorithm were of higher quality than the baseline state-of-the-art methods. Additionally, KMC was capable of obtaining results over larger and smaller datasets, especially for text data. It is also worth mentioning that in most scenarios, KMC jointly achieves the lowest entropy, largest purity and best normalized mutual information. Assuming a correct assignment of ground truth labels, these results suggest that the exception managing mechanism introduced by our proposal along with its accumulative instance-cluster weights succeed in the final assignment of problematic data objects.

In addition to attaining comparable results over most datasets, it seems interesting to notice that KMC achieves an entropy that is better by a half compared with the other methods on the following datasets: BBC-seg3, BBCSports-seg2, and BBCSports-seg4. Furthermore, the performance values attained by KMC over BBC-seg2, BBC-seg4, BBCSports-seg2, BBCSports-seg3 and BBCSports-seg4 either on Purity, normalized mutual information (or both) presents a positive difference of over ten percent compared with the second best method.

Notwithstanding the promising results obtained by KMC, it is also important to analyze particularly its performance on the Caltech dataset. In this scenario, our proposal is relatively far behind the two best methods in every measure. The values presented suggest that the extra refinements made over the co-association matrix by the winning methods provide additional insights for the consensus procedure that our proposal is unable to capture.

Along with the Caltech dataset, the Handwritten dataset also shows that KMC has a lower performance when dealing with non-text data, more specifically image data. A possible cause for this decrease in performance could be due to poor quality solutions initially found by the base method. Nevertheless, these two datasets were presented as unfavorable scenarios to KMC.

To conclude on the experimental section over real data, we can see that our proposed method has shown to be very competitive compared with other state-of-the-art methods, some of them corresponding to quite recently published works.

### 4.3. Empirical Analysis of the Stability of the Consensus Solution

The aim of this section is to empirically study how the quality of the consensus solution built by KMC is affected by the degree of discrepancy among the data views. To this end, we simulate several artificial multi-view datasets presenting different degrees of disagreement between the views, and we apply our method to them. In this way, we can sweep a range of discrepancy level values and assess the quality of the final ensemble solution independently of the base method used to build the initial solutions.

#### Procedure

We consider artificial datasets made of several views over the same data instances. Since we want to assess the influence of factors such as the number of clusters, the number of views, and the quality of the local partitions on our methods, we used the following methodology:

- Depending on the simulation, we generated multi-view data belonging to *k* clusters spread across eight views, and the matching ground-truth partitions.
- In *m* views out of eight, the partitions were altered with a degree of noise $p_{h}$ that corresponds to random changes in partition assignments to simulate varying qualities of local solutions.
- In the other partitions (not part of the *m* out of 8 altered partitions), a ration of only 5% alteration was applied.

**Figure 3:**
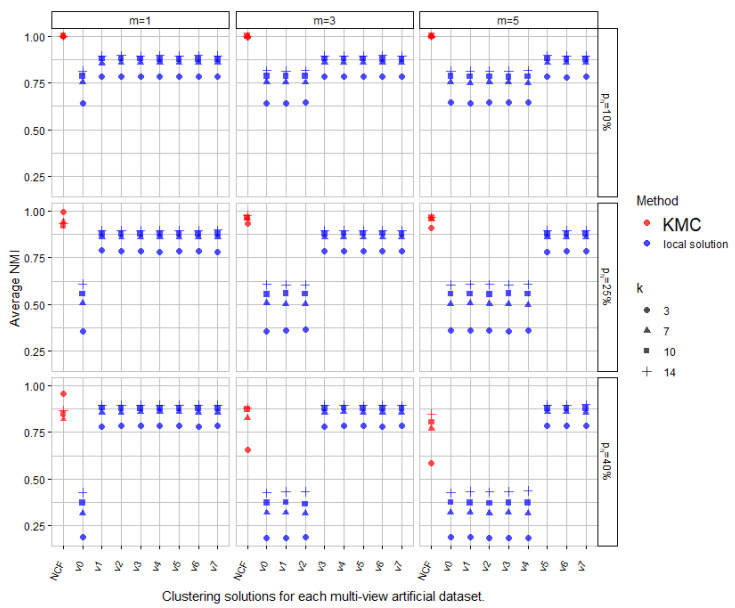
Average NMI values measured over all views plus the consensus solutions across all simulations.

During our experiments, we tried several combinations of number of clusters (3, 7, 10, and 14), number of altered views (1, 3 and 5 out of 8), and different ratios of alteration (10%, 25% and 40%). Finally, the average NMI was measured over all views, plus the consensus solutions across all simulations as depicted in Figure 3.

Figure 3 presents the quality of the attained results in terms of the NMI measure over several datasets generated under varying levels of agreement between the views. From the figure, we can see several things. As expected, the better results are achieved for configurations with less noisy views and a lower degree $p_{h}$ of noise alteration. When focusing on the analysis in the central plot, we observe that 3 out of 8 views show a larger level of disagreement regarding the ground truth partition (25%). However, we can also see that in many cases where the number of noisy views remains reasonable, KMC manages to achieve higher-quality consensus partitions in comparison to all the other views (seven configurations out of nine), almost regardless of the number of clusters we tested.

## 5. Conclusions and Future Works

In this paper, we presented KMC, a novel multi-view clustering fusion method relying on the notion of Kolmogorov complexity. This method is able to detect discrepancies and common points, and to merge partitions acquired from different algorithms or views. While minimum description length and Kolmogorov complexity have already been used in multi-view clustering contexts, the originality of our method lies in two points:

- Unlike in [7], which introduced the use of Kolmogorov complexity in a multi-view setting, we aim at a full merging of the clusters, and not just optimizing things locally.
- Unlike in [51], which is multi-view with merging, the optimization process to reduce these conflicts and merge partitions in a effective manner, we propose a new and improved algorithm to choose the merging order of the partitions, thus making our algorithm stable compared with earlier versions of the same technique.

Furthermore, our algorithm also uses techniques from unsupervised ensemble learning (merging partitions from several algorithms), and is applied to a multi-view clustering context (multiple representations of the same data objects).

We compared our method with algorithms from the field of unsupervised ensemble learning and multi-view cluster on several datasets from the literature. Despite strong differences in term of algorithm philosophies and original applications, we demonstrated that KMC attains a competitive consensus quality in relation to the state-of-the-art techniques. Its competitiveness coupled with the novelty it brings in terms of its core principle is an important contribution to the field of unsupervised ensemble learning, as it increases the variety of available methods, which is quite important in an unsupervised setting.

Finally, we also conducted an empirical study about how our method reacts under different noise conditions in a varying number of views, and the influence of the number of clusters. These results give a unique insight to the fine properties of the proposed algorithm, which has proved to be resilient to noise and very adaptive. This part of the study is very important, as it is unknown how other methods from the state of the art behave in similar conditions.

In our future works, we plan on focusing more on the theoretical properties of our algorithm that may be extracted from the empirical study. In particular, it would be interesting to have bounds based on levels of noises, but also to study the influence of clustering stability in local partitions and the role it may play in the merge result after our method.

## Figures and Tables

**Table 1:** Summary of all notations.

Notation	Meaning
$X=\{x_{1},x_{2}\ldots ,x_{N}\}$	The dataset of *N* objects split into the *M* views
*M*	The number of views
M	Turing machine or a computational clustering method
$\Pi^{\left(M\right)}$	The partition built by method M
$\Pi^{i}$	The partition in view *i*
$K^{i}$	The number of clusters in view *i*
$K_{a}^{i}$	The *a*-th cluster in view *i*
$L^{i}:X^{i}\to \left[1\ldots K^{i}\right]$	The function mapping any element of view *i* to a cluster of this view
$\Omega^{ij}$	The mapping matrix from view *i* to view *j*
$\omega_{a,b}^{ij}$	The percentage of elements from $K_{a}^{i}$ in that also belong to $K_{b}^{j}$
$\Phi^{j}\left(K_{a}^{i}\right)$	The maximum agreement cluster for $K_{a}^{i}$ in view *j*
$K_{\cdot }^{i}\left(x\right)$	Objects belonging to the same max agreement cluster than *x* in $\Pi^{i}$
$K(\Pi^{i}|\Pi^{j})$	Kolmogorov complexity of $\Pi^{i}$ knowing $\Pi^{j}$, see Equation (5)
$\epsilon_{i,j}$	The error list when mapping $\Pi^{i}$ to $\Pi^{j}$, see Equation (6)
$\xi_{k}$	The exception set for any partition $\Pi^{k}$ (points marked in $\epsilon_{i,k}$ or $\epsilon_{k,j}$)
$\mu_{p}^{k}\left(x\right)$	Membership weight of point $x\in \xi_{k}$ to a cluster $K_{p}^{k}$, see Equation (7)
*W*	list of weight for all partitions
Q	List of all previous merge exception ($\xi_{k}$)
$\Psi [K_{p}^{i}]$	consensus assignments made for each cluster $K_{p}^{i}$, see Algorithm 2

**Table 2:** Overall description of the real data collections.

Dataset	#Doc	#Views
3Sources	169	3
BBC-seg2	2012	2
BBC-seg3	1268	3
BBC-seg4	685	4
BBCSports-seg2	544	2
BBCSports-seg3	282	3
BBCSports-seg4	116	4
Handwritten	2000	6
Caltech	2386	6

**Table 3:** Performance results of each method assessed through several external quality measures. Best results are in bold, and second best are underlined.

Dataset	Method	Entropy	Purity	NMI
3Sources	CSPA [28]	0.4807	0.6864	0.4314
	HGPA [28]	0.4362	0.6805	0.4789
	MCLA [28]	0.4990	0.6746	0.4202
	ECPCS-MC [46]	0.3465	0.8047	0.5047
	ECPCS-HC [46]	0.4008	0.7456	0.5301
	MDEC-BG [45]	0.3431	0.7929	0.4899
	MDEC-HC [45]	0.3471	0.8047	0.5503
	MDEC-SC [45]	0.3406	0.7929	0.4990
	KMC	0.3776	0.6935	0.5512
BBC-seg2	CSPA	0.3472	0.8226	0.5350
	HGPA	0.3957	0.7639	0.4968
	MCLA	0.3481	0.8221	0.5404
	ECPCS-MC	0.3413	0.8236	0.6072
	ECPCS-HC	0.2914	0.8673	0.6522
	MDEC-BG	0.3188	0.8405	0.5729
	MDEC-HC	0.2785	0.8703	0.6229
	MDEC-SC	0.2816	0.8743	0.5967
	KMC	0.1525	0.9488	0.8468
BBC-seg3	CSPA	0.3430	0.8226	0.5290
	HGPA	0.5489	0.6467	0.3603
	MCLA	0.3995	0.7989	0.4852
	ECPCS-MC	0.3647	0.7934	0.5336
	ECPCS-HC	0.3715	0.8115	0.6088
	MDEC-BG	0.3048	0.8573	0.5683
	MDEC-HC	0.3497	0.8375	0.5996
	MDEC-SC	0.3651	0.7997	0.5131
	KMC	0.1517	0.9519	0.8453
BBC-seg4	CSPA	0.4327	0.7182	0.4308
	HGPA	0.4689	0.6978	0.4024
	MCLA	0.4643	0.7518	0.4100
	ECPCS-MC	0.4145	0.7869	0.4639
	ECPCS-HC	0.4708	0.7066	0.4646
	MDEC-BG	0.3490	0.8321	0.5126
	MDEC-HC	0.4324	0.7401	0.4719
	MDEC-SC	0.3899	0.7825	0.4744
	KMC	0.2657	0.8336	0.7016
BBCSports-seg2	CSPA	0.3897	0.7463	0.4647
	HGPA	0.4729	0.6912	0.3954
	MCLA	0.9406	0.3548	0.0000
	ECPCS-MC	0.3302	0.8125	0.5931
	ECPCS-HC	0.3290	0.8143	0.5984
	MDEC-BG	0.3486	0.7831	0.5097
	MDEC-HC	0.3263	0.8088	0.5452
	MDEC-SC	0.3350	0.8070	0.5278
	KMC	0.1237	0.9540	0.8674
BBCSports-seg3	CSPA	0.4983	0.6560	0.3434
	HGPA	0.5507	0.6241	0.3000
	MCLA	0.5247	0.6489	0.3327
	ECPCS-MC	0.5336	0.6596	0.3682
	ECPCS-HC	0.5184	0.6099	0.4122
	MDEC-BG	0.4756	0.7021	0.3697
	MDEC-HC	0.4821	0.7092	0.3806
	MDEC-SC	0.4842	0.6986	0.3592
	KMC	0.2595	0.8227	0.6802
BBCSports-seg4	CSPA	0.7382	0.4569	0.1458
	HGPA	0.7915	0.4052	0.1004
	MCLA	0.7284	0.4741	0.1608
	ECPCS-MC	0.7668	0.4397	0.1249
	ECPCS-HC	0.7682	0.3966	0.1488
	MDEC-BG	0.7363	0.4828	0.1516
	MDEC-HC	0.7499	0.4741	0.1413
	MDEC-SC	0.7770	0.4655	0.1158
	KMC	0.3087	0.7854	0.6427
Handwritten	CSPA	0.2040	0.8890	0.7960
	HGPA	0.3216	0.7570	0.6807
	MCLA	0.2811	0.7885	0.7196
	ECPCS-MC	0.1838	0.8795	0.7376
	ECPCS-HC	0.2031	0.8520	0.7870
	MDEC-BG	0.1584	0.8915	0.7414
	MDEC-HC	0.1471	0.8935	0.7708
	MDEC-SC	0.1652	0.8890	0.7371
	KMC	0.2470	0.8230	0.7250
Caltech	CSPA	0.4003	0.6639	0.4960
	HGPA	0.4415	0.5985	0.4450
	MCLA	0.3918	0.6601	0.5118
	ECPCS-MC	0.2703	0.7531	0.5717
	ECPCS-HC	0.2497	0.7695	0.6591
	MDEC-BG	0.2166	0.8139	0.5914
	MDEC-HC	0.2463	0.7930	0.6222
	MDEC-SC	0.2229	0.7963	0.5989
	KMC	0.4540	0.6500	0.4320

## Data Availability

Sources can be found at https://github.com/jfzo/Multiview-clustering/releases/tag/Journal, last accessed on February 2023.

## Abbreviations

The following abbreviations are used in this manuscript:

CSPA	Cluster-based Similarity Partitioning
ECPCS	Ensemble Clustering via fast Propagation of Cluster-wise Similarities
HGPA	Hyper Graph Partitioning
KMC	Kolmogorov-based Multi-view Clustering
MCLA	Meta Clustering
MDEC	Multidiversified Ensemble Clustering
NMI	Normalized Mutual Information
